# Indications for endodontic surgery in private practice and temporal trends before and after adoption of laser-activated irrigation: a retrospective practice-based study

**DOI:** 10.1007/s00784-026-07199-7

**Published:** 2026-10-03

**Authors:** David Han, Christopher Chun, Catherine Kim, Laurel Martinez, Jooyoung Song

**Affiliations:** Apical Endodontics, 28212 Kelly Johnson Pkwy, #290, Valencia, CA 91355 USA

**Keywords:** Endodontic Microsurgery (EMS), Laser-Activated Irrigation (LAI), Er,Cr:YSGG laser, Before-Laser Treatment (BLT), After-Laser Treatment (ALT)

## Abstract

**Objectives:**

To characterize indications for endodontic microsurgery (EMS) and compare the relative proportion of these indications across two calendar periods before and after the practice’s adoption of laser-activated irrigation (LAI).

**Materials and methods:**

A retrospective time-period comparison of consecutive EMS cases (*n* = 1,672) treated by six endodontists (April 2019–August 2025) was analyzed. Two periods were prespecified based exclusively on the date of surgery: Before-Laser Treatment (BLT) and After-Laser Treatment (ALT) following implementation of an Er, Cr: YSGG intracanal LAI protocol (August 2022). Each surgical tooth was assigned one or two predefined reasons. Proportions were compared between periods using Generalized Estimating Equations (GEE) adjusting for patient clustering, demographics, tooth class, and provider.

**Results:**

The most frequent indications overall were large post (22.2%), uncleaned accessory canal (21.8%), and excessive calcification (14.4%). In adjusted models, the proportion of EMS cases attributed to uncleaned accessory canals decreased from 26.6% (204/767) in BLT to 17.7% (160/905) in ALT (adjusted OR = 0.49; 95% CI 0.37 to 0.65; *P* < .001). Conversely, the proportion of EMS cases attributed to excessive calcification increased from 10.8% (83/767) to 17.3% (157/905) (adjusted OR = 1.65; 95% CI 1.21 to 2.25; *P* = .002).

**Conclusions:**

In this practice-based case series, the ALT period was associated with a lower proportion of EMS cases performed for uncleaned accessory canals. By contrast, the proportion of EMS cases attributed to excessive calcification was higher in the ALT period. Causal inference regarding LAI effectiveness is limited by the time-period design and the lack of non-surgical denominator.

**Clinical relevance:**

Following the adoption of laser-activated irrigation (LAI) in non-surgical endodontics, uncleaned accessory anatomy constituted a smaller relative share of subsequent microsurgical cases. Conversely, clinicians should anticipate a continued substantial burden of calcification- and post-related surgical challenges.

## Introduction

Endodontic microsurgery (EMS) encompasses a spectrum of surgical procedures—including root-end apicoectomy, root amputation, intentional replantation, and external root resorption repair—each with distinct clinical applications. While intentional replantation involves the extraction of the tooth, it is operationally categorized as an endodontic microsurgical procedure in this cohort; following atraumatic extraction, all excavation of granulation tissue on the root surface, root resection, retropreparation, and retrofilling are executed under the careful view of a high-powered operating microscope while utilizing copious irrigation with Hanks’ Balanced Salt Solution (HBSS) over a sterile pan. The literature indicates that all four types of EMS can yield favorable outcomes, provided that case selection is appropriate and clinical circumstances are carefully considered [1–4]. Although conventional non-surgical endodontic therapy remains the primary modality for treating pulpal and periradicular disease, certain cases necessitate surgical intervention. Modern EMS has demonstrated higher success rates than traditional approaches, largely owing to advances in ultrasonic root-end preparation, biocompatible filling materials, refined microsurgical instrumentation, and enhanced magnification and illumination [5, 6]. Reported indications for surgery include persistent extraradicular infection, procedural mishaps such as instrument separation or perforation, untreated anatomy, and root fractures [7–10]. Existing literature has largely focused on isolated indications; however, no known studies to date have comprehensively analyzed the full spectrum of reasons for EMS in a single cohort. This detailed understanding of the common and uncommon indications is important for determining the necessity for surgery, refining treatment protocols, and improving patient care.

Concurrently, a growing body of evidence supports the use of adjunctive technologies to enhance the effectiveness of conventional non-surgical endodontic procedures. Among these, laser-activated irrigation (LAI) has emerged as a promising method to enhance disinfection within the complex root canal system and to reduce postoperative pain [11–15]. However, the potential long-term impact of LAI on the subsequent need for EMS has not been clearly established in a clinical setting. To date, no studies have evaluated whether the availability of LAI during non-surgical treatment is associated with a change in the relative distribution of indications for EMS.

The purpose of this retrospective practice-based study was twofold. First, to characterize the specific indications for EMS by quantifying the frequency of 15 reasons in a large private practice cohort. Second, to test our a priori hypothesis that the distribution of EMS indications would differ across pre- and post- LAI periods for two specific mechanically related indications (uncleaned accessory canal and excessive calcification), acknowledging that this time-period comparison cannot establish causality.

## Materials and methods

This retrospective observational study is reported in accordance with the STROBE guideline; the completed checklist is provided as a supplementary file and the study flow is detailed in Fig. 1.

**Fig. 1:**
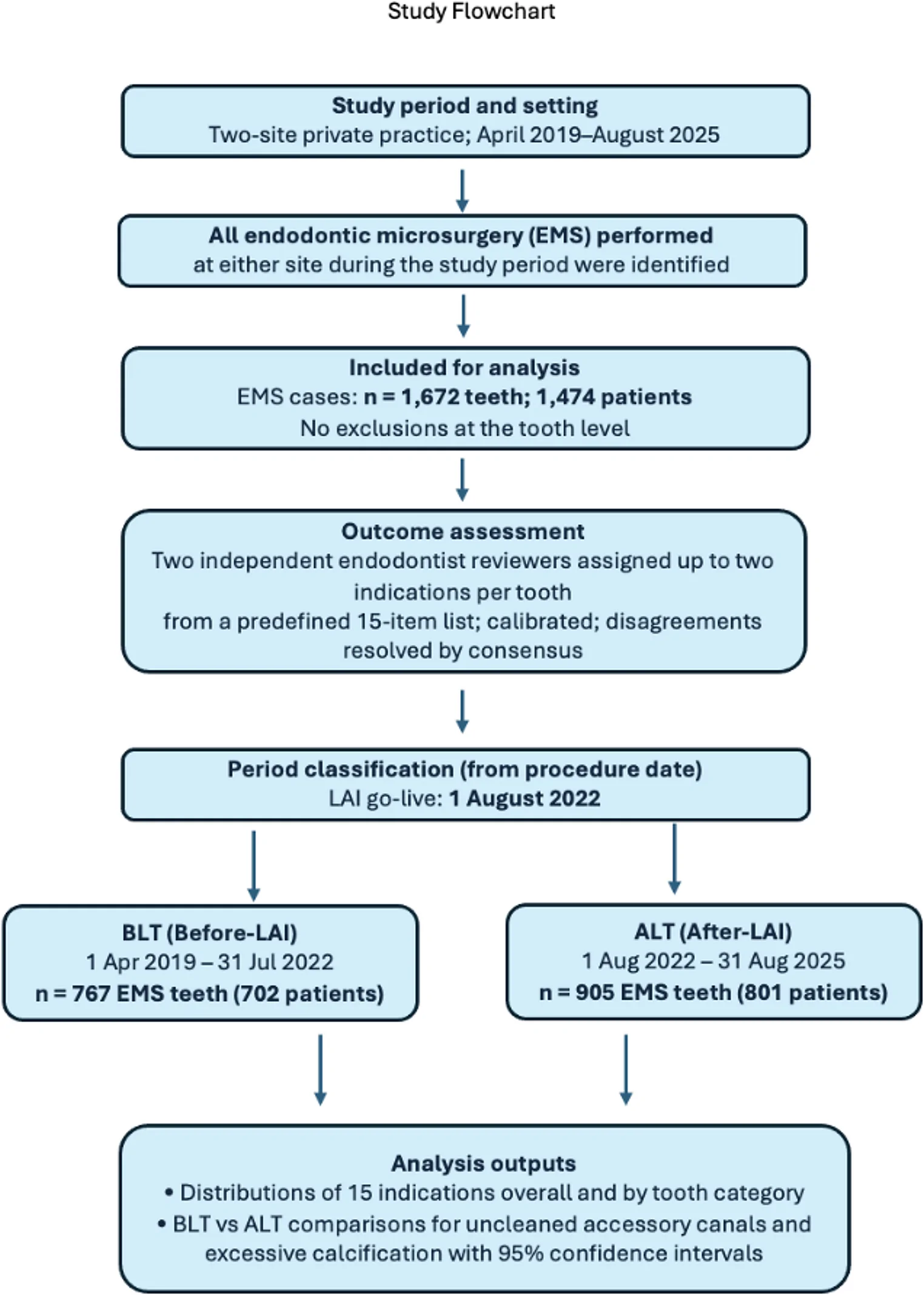
Study flow diagram. Two-site private practice (April 2019–August 2025). All EMS procedures were identified; *n* = 1,672 teeth (1,474 patients) were included with no tooth-level exclusions. Period classification derived from procedure date (Before–LAI, 1 Apr 2019–31 Jul 2022; After–LAI, 1 Aug 2022–31 Aug 2025). Because a subset of patients (*n* = 29) underwent separate surgeries in both the BLT and ALT periods, the sum of the period-specific patient counts naturally exceeds the total unique patient count of the overall cohort

### Study cohort and setting

This study analyzed de-identified clinical data from a multi-site private endodontic practice located in a major metropolitan area. The cohort consisted of 1,474 patients who underwent endodontic microsurgery (EMS) on 1,672 teeth between April 1, 2019, and August 31, 2025. Data were divided into two groups based on the implementation of a specific disinfection protocol: -Before-Laser Treatment (BLT): April 1, 2019, to July 31, 2022, using conventional chemomechanical irrigation. -After-Laser Treatment (ALT): August 1, 2022, to August 31, 2025, following the routine adoption of laser-activated irrigation (LAI). The three-month difference in the study period lengths between the two groups is attributed to the COVID-19 pandemic with a one-month office closure and a subsequent two-month period where no endodontic surgeries were performed due to an overabundance of caution taken to minimize the transfer of COVID-19. No a priori sample-size calculation was performed; the cohort represents a consecutive census of all eligible EMS cases during the study periods.

Importantly, period classification was based exclusively on the date of the surgical procedure. Because the cohort includes external surgical referrals and teeth whose index non-surgical treatments were performed years prior, LAI exposure during the initial treatment was not verified at the individual-tooth level. Therefore, the BLT and ALT groups represent a calendar-period comparison rather than a confirmed exposure-based cohort.

### Clinical protocols

All procedures were performed by six endodontists who received standardized specialist training. The non-surgical endodontic protocol was uniform across all clinicians. After anesthesia and dental-dam isolation, an access cavity was prepared and working length established with an electronic apex locator and confirmed radiographically. Initial glidepath preparation was performed with 0.02-taper stainless-steel hand files, followed by preparation to a 0.20 mm tip size at the working length. Mechanical instrumentation then proceeded using a crown-down approach with 0.04- or 0.06-taper nickel–titanium rotary files under copious irrigation with 4% sodium hypochlorite. In the standard chemomechanical protocol—which defined the BLT period—4% sodium hypochlorite served as the primary irrigant throughout instrumentation. Typically, smear-layer removal was performed at the end of preparation using 17% EDTA followed by a final 4% sodium hypochlorite rinse. However, in selected situations—such as the presence of pulp stones, heavy smear layer, or the need to enhance visualization of canal orifices on the chamber floor—17% EDTA was used earlier in the treatment. After complete preparation, obturation was performed with gutta-percha cones and bioceramic sealer using a downpack-and-backfill technique.

The protocol in the ALT period utilized the standard mechanical and shaping procedures described above, with LAI serving as the final disinfection step. At the completion of full canal instrumentation, LAI was performed using the EdgePRO™ (Brasseler USA) Er, Cr: YSGG laser (2780 nm) equipped with a radial-firing fiber tip (e.g., 200–300 μm diameter, depending on canal curvature). The system utilized manufacturer-recommended micro-pulse activation with settings of 0.1–0.15 W average power, 5–7.5 mJ per pulse, and a 20 Hz frequency. Standard laser safety precautions, including specialized protective eyewear for the patient and surgical team, alongside continuous high-volume evacuation, were strictly followed. The unactivated fiber tip was inserted to the middle third of the canal (approximately 2 mm short of the working length) ensuring no binding. Activation consisted of slow withdrawal with circumferential sweeping motions for 5 s per canal. A specific Hybrid Technique Protocol was utilized with the device air/water spray disabled. Each activation cycle utilized approximately 2 mL of fresh irrigant replenished via a side-vented needle. The sequence consisted of a 5-second activation in saline, followed by replenishment and a 5-second activation in 17% EDTA, and concluding with a final replenishment and 5-second activation in 4% sodium hypochlorite. Notably, the LAI system was occasionally utilized earlier in the protocol, such as in situations where canals were severely calcified and patency could not be achieved; in these instances, LAI with 17% EDTA was employed before final canal preparation to facilitate the negotiation of micro-canals.

The sampling frame comprised all EMS cases; non-surgical endodontic treatments were outside the analytic scope because outcomes were defined exclusively as indications for EMS, and our period contrasts are within-EMS proportions.

### Surgical indications and decision making

EMS in this study were performed under operating microscope magnification and illumination using modern microsurgical instrumentation, bioceramic root-end filling material, guided tissue regeneration when indicated, and Cone Beam Computed Tomography (CBCT) imaging. The clinicians share common diagnoses and treatment philosophies, as all were trained at the same specialty/residency program. The endodontists followed a uniform, stepwise approach: Once endodontic disease is diagnosed, non-surgical endodontic therapy or retreatment is first considered. EMS is considered only when non-surgical treatment is infeasible or unlikely to succeed—for example, when root integrity would be compromised (e.g., a large cast post extending > two-thirds of canal length), when canal patency cannot be achieved despite appropriate attempts and the patient has persistent symptoms and/or a periapical radiolucency, or when external root resorption (apical or along lateral external root surface) cannot be excavated and predictably restored non-surgically. When non-surgical retreatment has been unsuccessful or is not possible and EMS is judged less predictable or unsafe due to anatomic risk —(e.g., palatal root of maxillary second molars close to the greater palatine vessels; mandibular second molars with proximity to the inferior alveolar nerve and thick buccal cortical bone), intentional replantation is considered provided key criteria are met—non-divergent root morphology amenable to atraumatic extraction and replantation and a stable periodontal status.

### Data collection

The study protocol involved a retrospective review where patient charts were de-identified and anonymized prior to data extraction to ensure no identifiable private information was used in the analysis. Data collected included the patient’s age and gender, the tooth number, the specific reason for endodontic microsurgery, the type of surgery, and the date of the procedure. Preoperative CBCT imaging and intraoperative biopsy (when adequate tissue could be curetted per American Association of Endodontists’ guidelines) were standard clinical protocols; however, exact execution varied due to real-world clinical constraints (e.g., patient financial limitations, non-retrievable cohesive tissue) (Tables 1, 2, 3 and 4). As a result, exact case-by-case imaging and biopsy frequencies were not tabulated. Operational definitions for the assignment of selected specific indications are detailed in Table 5. The types of endodontic microsurgery included root-end apicoectomy, root amputation, intentional replantation, and external root resorption repair. The specific reasons for endodontic microsurgery included: (1) Apical cementicle, (2) Apical cyst, (3) Endodontic-periodontic infection, (4) Excessive calcification, (5) External root resorption, (6) Extrusion, (7) Extraradicular pathosis/Actinomycosis, (8) Failed apical surgery, (9) Large post, (10) Palatal gingival groove, 11) Root fracture, 12) Separated instrument, 13) Transportation/perforation, 14) Uncleaned accessory canal, and 15) Unknown etiology. The “Unknown etiology” category was used for cases where endodontic microsurgery was performed despite all root canal obturations reaching the apex (apices) and no known accessory canals (including delta canals, lateral canals, and isthmi) being visualized during non-surgical nor surgical treatments. In these instances, surgery was necessitated by either persistent symptoms and/or a persistent growth in the periapical radiolucency. In addition, biopsy results for these cases were negative for apical cyst and extraradicular pathosis/Actinomycosis.

**Table 1:** Baseline demographic and clinical characteristics of the study cohort by calendar period (*N* = 1,672 teeth; 1,474 patients)*

Variable	Total cohort (*n* = 1672)	BLT period (*n* = 767)	ALT period (*n* = 905)
Patient Age (Years)			
Mean	56.4	56.5	56.2
Median	58	58	58
Patient Sex (Per Tooth)., n (%)			
Female	969 (58.0%)	456 (59.5%)	513 (56.7%)
Male	703 (42.0%)	311 (40.5%)	392 (43.3%)
Tooth Category, n (%)			
Maxillary Anteriors	306 (18.3%)	143 (18.6%)	163 (18.0%)
Maxillary Premolars	305 (18.3%)	160 (20.9%)	145 (16.0%)
Maxillary Molars	559 (33.4%)	255 (33.3%)	304 (33.6%)
Mandibular Anteriors	97 (5.8%)	43 (5.6%)	54 (6.0%)
Mandibular Premolars	55 (3.3%)	23 (3.0%)	32 (3.5%)
Mandibular Molars	350 (20.9%)	143 (18.6%)	207 (22.9%)
Type of Surgery, n (%)			
Root-end apicoectomy	1,573 (94.1%)	720 (93.9%)	853 (94.3%)
External root resorption repair	64 (3.8%)	28 (3.6%)	36 (3.9%)
Root amputation	21 (1.3%)	14 (1.8%)	7 (0.8%)
Intentional replantation	14 (0.8%)	5 (0.7%)	9 (1.0%)

*The denominator differs between surgery type (1,672 teeth) and sex distribution (1,474 patients) because some patients underwent microsurgery on more than one tooth

**Table 2:** Frequency and distribution of indications for endodontic surgery by calendar period

Reasons for EMS	Total (*n* = 1672) n (%)	BLT (*n* = 767) n (%)	ALT (*n* = 905) *n* (%)
Apical Cementicle	4 (0.2%)	1 (0.1%)	3 (0.3%)
Apical Cyst	206 (12.3%)	125 (16.3%)	81 (9.0%)
Endodontic – Periodontic infection	5 (0.3%)	4 (0.5%)	1 (0.1%)
Excessive Calcification	240 (14.4%)	83 (10.8%)	157 (17.3%)
External Root Resorption	107 (6.4%)	51 (6.6%)	56 (6.2%)
Extraradicular Pathosis/Actinomycosis	46 (2.8%)	23 (3.0%)	23 (2.5%)
Extrusion	63 (3.8%)	29 (3.8%)	34 (3.8%)
Failed apical surgery	32 (1.9%)	12 (1.6%)	20 (2.2%)
Large Post	372 (22.2%)	170 (22.2%)	202 (22.3%)
Palatal Gingival Groove	1 (< 0.1%)	0 (0.0%)	1 (0.1%)
Root Fracture	37 (2.2%)	22 (2.9%)	15 (1.7%)
Separated Instrument	43 (2.6%)	28 (3.7%)	15 (1.7%)
Transportation/Perforation	143 (8.6%)	55 (7.2%)	88 (9.7%)
Uncleaned Accessory Canal	364 (21.8%)	204 (26.6%)	160 (17.7%)
Unknown Etiology	125 (7.5%)	38 (5.0%)	87 (9.6%)

*Percentages represent the proportion of EMS cases assigned the indication. Column totals exceed 100% because up to two indications could be assigned per tooth (116 teeth had dual indications)—78 from BLT period and 38 from ALT period

**Table 3:** Adjusted multivariable GEE models for the odds of indication assignment among EMS cases*

Variable	Uncleaned accessory canal		Excessive calcification	
	Adjusted OR (95% CI)	*P* Value	Adjusted OR (95% CI)	*P* Value
Period				
BLT (ref)	1.00	-	1.00	-
ALT	0.49 (0.37 to 0.65)	< 0.001	1.65 (1.21 to 2.25)	0.002
Age (Years)	0.99 (0.99 to 1.00)	0.214	1.01 (1.00-1.03)	0.013
Sex				
Female (ref)	1.00	-	1.00	-
Male	0.88 (0.67 to 1.16)	0.367	0.95 (0.71 to 1.27)	0.722
Tooth Category				
Maxillary Anteriors (ref)	1.00	-	1.00	-
Maxillary Premolars	9.64 (3.35 to 27.72)	< 0.001	2.21 (1.21 to 4.06)	0.010
Maxillary Molars	34.32 (12.33 to 95.49)	< 0.001	5.38 (3.15 to 9.21)	< 0.001
Mandibular Anteriors	0.84 (0.09 to 7.79)	0.878	0.30 (0.07 to 1.38)	0.123
Mandibular Premolars	1.60 (0.18 to 14.64)	0.677	0.55 (0.12 to 2.46)	0.431
Mandibular Molars	68.57 (24.48 to 192.10)	< 0.001	1.95 (1.07 to 3.55)	0.029
Practice Site				
Site A (ref)	1.00	-	1.00	-
Site B	0.97 (0.72 to 1.30)	0.825	0.92 (0.66 to 1.26)	0.589
Surgeon				
Surgeon 1 (ref)	1.00	-	1.00	-
Surgeon 2	1.00 (0.61 to 1.63)	0.994	1.44 (0.91 to 2.28)	0.122
Surgeon 3	1.41 (0.94 to 2.11)	0.094	0.96 (0.58 to 1.60)	0.883
Surgeon 4	1.86 (1.14 to 3.03)	0.013	1.95 (1.14 to 3.33)	0.014
Surgeon 5	0.29 (0.04 to 2.09)	0.221	1.95 (0.7 to 5.36)	0.195
Surgeon 6	1.43 (0.52 to 3.96)	0.486	2.25 (0.94 to 5.42)	0.069

*Models adjusted for patient age, sex, tooth class, surgeon, and practice site

**Table 4:** Top three indications for endodontic microsurgery by tooth category (percentages calculated within each category; some totals exceed 100% because up to two reasons could be assigned per tooth)

Tooth category	1st reason (*n*, %)	2nd reason (*n*, %)	3rd reason (*n*, %)
Maxillary Anteriors	Large Post; 140 (45.8%)	Apical Cyst; 50 (16.4%)	External Root Resorption; 30 (9.9%)
Mandibular Anteriors	Large Post; 34 (35.8%)	External Root Resorption; 20 (21.1%)	Unknown etiology; 9 (9.5%)
Maxillary Premolars	Large Post; 120 (39.3%)	Uncleaned accessory canal; 37 (12.0%)	Apical Cyst; 30 (9.7%)
Mandibular Premolars	Large Post; 18 (32.7%)	External Root Resorption; 15 (27.3%)	Unknown etiology; 9 (16.4%)
Maxillary Molars	Excessive Calcification; 150 (26.9%)	Uncleaned accessory canal; 150 (26.9%)	Transportation/perforation; 71 (12.7%)
Mandibular Molars	Uncleaned accessory canal; 152 (43.4%)	Large Post; 53 (15.0%)	Excessive Calcification; 50 (14.2%)

**Table 5:** Operational criteria and diagnostic evidence for selected surgical indications

Indication	Operational definition & required diagnostic evidence
Uncleaned accessory canal	Identified intraoperatively during surgery via high-power surgical microscopy (often aided by methylene blue staining) after root-end resection. Defined as the presence of untreated lateral canals, isthmi, or apical ramifications containing necrotic debris or previous filling material that were not completely debrided during prior orthograde treatment.
Excessive calcification	Confirmed preoperatively via CBCT and/or intraoperatively during non-surgical endodontic treatment. Defined as severe pulp canal obliteration (PCO) preventing orthograde negotiation to the apical terminus despite the use of magnification, ultrasonics, and chelating agents, resulting in persistent pathosis.
Apical cyst	Defined as a pathological cavity lined by epithelium at the root apex. Assigned exclusively upon definitive postoperative histopathologic confirmation of the biopsied lesion. Note: This classification encompasses both true and pocket cysts, as routine surgical biopsy cannot reliably distinguish between the two.
Extraradicular pathosis / Actinomycosis	Confirmed exclusively via histopathologic examination of periapical biopsy tissue demonstrating characteristic bacterial colonies (e.g., *Actinomyces* species exhibiting sulfur granules or branching filamentous structures) within the extraradicular tissue.
Root fracture	Identified preoperatively (via CBCT demonstrating classic bone loss patterns like J-shaped lesions) or definitively visualized intraoperatively under the surgical microscope as a distinct structural crack or fracture line extending into the root dentin.
Endodontic-periodontic infection	Diagnosed preoperatively and intraoperatively based on the presence of a deep periodontal pocket communicating with a periapical lesion (retrograde periodontitis) or a primary periodontal lesion secondarily infecting the pulp, where both endodontic and periodontal surgical intervention (e.g., bone grafting/guided tissue regeneration) were required simultaneously.

Each EMS case was assigned one or two indications from the 15-item list. When two applicable indications were present, both were recorded; 116 teeth (6.9% of the cohort) had dual indications. For example, for tooth #19, the mesiobuccal canal exhibited a non-negotiable excessive calcification while the distal canal contained a separated instrument in the apical third around a distal curvature; therefore, the indications assigned were “excessive calcification” and “separated instrument.” For all descriptive and regression analyses, each of the 15 indications was treated as a separate, independent binary variable (“present” or “absent” for that tooth). Because our statistical models evaluated the odds of a specific indication being assigned rather than modeling a single mutually exclusive categorical outcome, the presence of dual-reason teeth did not violate model assumptions or bias the period-level differences.

Two endodontists reviewed each surgical record to assign up to two indications per tooth from the predefined list (Table 5). The reviewers were not blinded to the calendar period. Rather than independent blinded scoring followed by a formal inter-rater reliability assessment (e.g., Kappa statistic), assignments were made through direct clinical calibration and consensus discussion and the final dataset was tabulated for analysis. Multivariable logistic regression models were fitted to estimate the adjusted odds of a specific indication being assigned among teeth undergoing EMS (outcome = indication present/absent). To account for non-independence due to patients contributing multiple teeth, patient-level clustering was addressed using Generalized Estimating Equations (GEE) with robust standard errors. The models included calendar period (ALT vs. BLT), patient age, sex, tooth class, practice site, and surgeon code as fixed-effect covariates. Collinearity was assessed via variance inflation factors (VIF), and there were no missing data for the included covariates.

### Statistical analysis

Proportions were compared using Pearson’s χ² test with two-sided α = 0.05; 95% confidence intervals were calculated by the Wald method. When expected counts were < 5, Fisher’s exact test was used as a sensitivity check. Analyses were performed in R, version 4.4.1 (R Foundation for Statistical Computing, Vienna, Austria). Descriptive statistics summarized patient demographics, the distribution of reasons for surgery, and procedure types. Because a tooth could contribute up to two indications, percentages for each reason were computed as occurrences divided by the total number of surgeries and may therefore exceed 100%. At most two indications were assigned per tooth. The number of teeth having two reasons for surgery was 116 out of 1,672 (6.9%). As an a priori specificity check against secular confounding, we analyzed “negative-control” indications (Large post; Root fracture). These were selected because they represent structural or mechanical failures that are biologically independent of the intracanal irrigation protocol, and therefore their relative proportions should theoretically remain stable across periods. BLT vs. ALT differences for these outcomes using Pearson’s χ² with two-sided α = 0.05 and 95% confidence intervals (CIs) were tested.

### Ethics statement

This retrospective study analyzed fully de-identified clinical records and did not involve patient contact. The project was formally determined to be exempt from ongoing human-subjects review by the WCG Institutional Review Board under 45 CFR 46.104(d)(4) for secondary research of existing data. Furthermore, the IRB granted a full waiver of HIPAA privacy authorization under 45 CFR 164.512(i), as the research could not practicably be conducted without the waiver and involved no more than minimal risk to privacy (IRB Study No.: 1401656; IRB Pr. No.: 20254353). The study adhered to the Declaration of Helsinki.

## Results

Analysis of patient demographics revealed a slight female predominance in the surgical cohort, with 57.7% of patients being female and 42.3% male. The overall mean age of patients undergoing endodontic microsurgery was 56.4 years (SD:14.8), with a median age of 58 years. In the BLT period, the mean age was 56.5 years (SD: 15.0) (median: 58), while in the ALT period, the mean age was 56.2 years (SD: 14.6) (median: 58). Among the 1,672 teeth treated surgically, the most common procedure was root-end apicoectomy, followed by external root resorption repair, root amputation, and intentional replantation (Table 1).

The reasons for endodontic microsurgery were analyzed in three sections. First, for the entire six-year study period, and then separately for the BLT and ALT groups. Representative cases of each reason for endodontic microsurgery are shown radiographically and/or with photos and/or with CBCT scan images (Fig. 2).

**Fig. 2:**
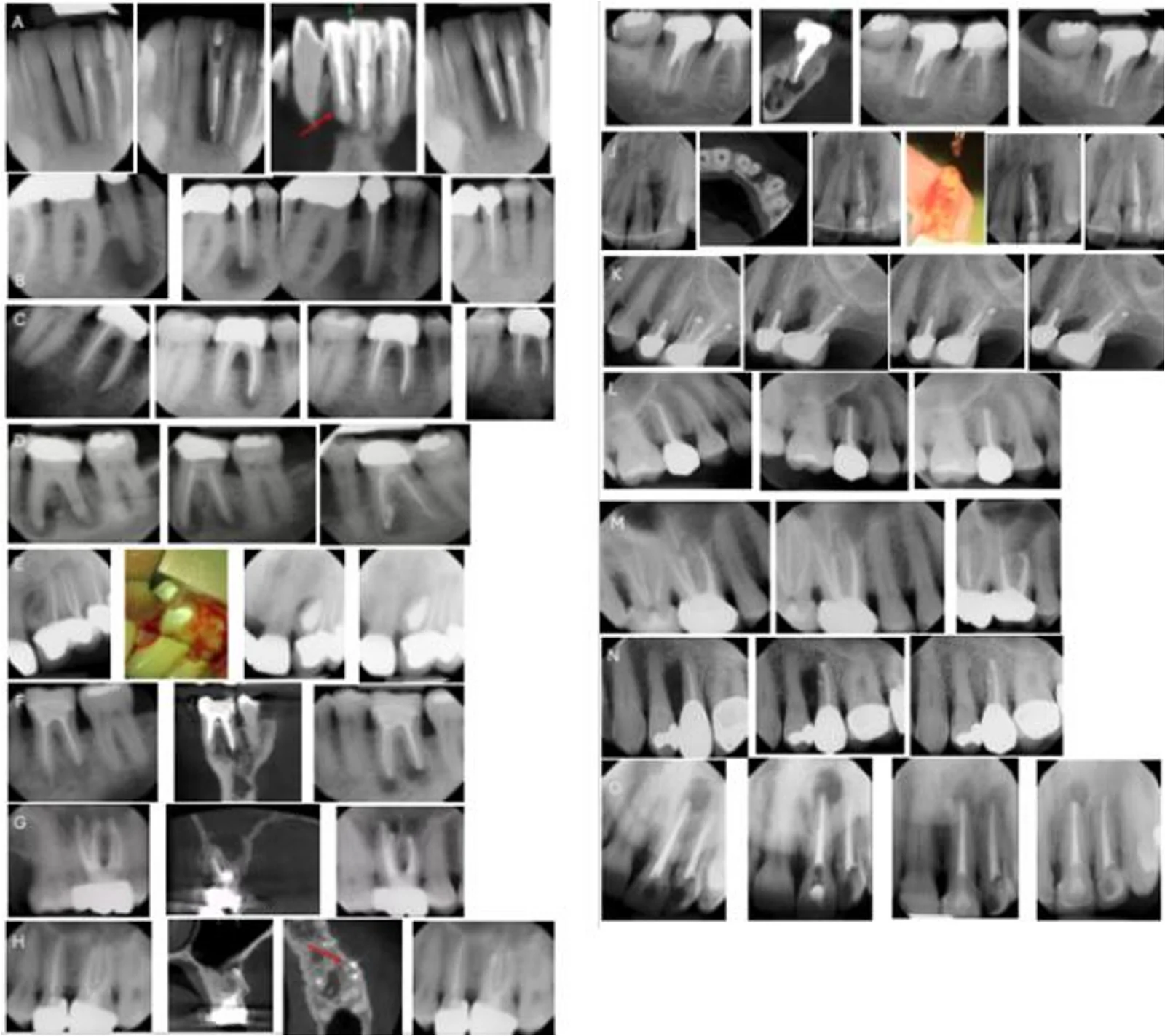
Representative examples for each of the 15 reasons for endodontic microsurgery. For each case, the radiograph prior to the surgical procedure is shown, along with a radiograph after the microsurgery and, when appropriate, postoperative non-surgical endodontic therapy radiographs, CBCT images and/or clinical photos and/or follow-up radiographs. (**A**) Apical Cementicle, arrow pointing to distal apical cementicle on root surface of tooth #25. (**B**) Apical cyst. (**C**) Endodontic-Periodontic infection. (**D**) Excessive calcification. (**E**) External root resorption (**F**) Extrusion (**G**) Extraradicular pathosis/Actinomycosis. (**H**) Failed apical surgery, Arrow pointing to missed, calcified MB2. (**I**) Large post. (**J**) Palatal gingival groove, Tooth #10, intentional replantation performed to repair deep lingual groove. (**K**) Root fracture, (**L**) Separated instrument. (**M**) Transportation/perforation. (**N**) Uncleaned accessory canal. (**O**) Unknown etiology, Tooth #8, despite non-surgical endodontic treatment and retreatment, symptomatic with persistent periapical radiolucency

The arch and tooth-class distribution of EMS-treated teeth is summarized in Fig. 3 (percentages calculated from the total cohort, *n* = 1,672). Maxillary molars comprised the largest share (559/1,672, 33.4%), followed by mandibular molars, maxillary premolars, maxillary anteriors, mandibular anteriors and lastly, mandibular premolars.

**Fig. 3:**
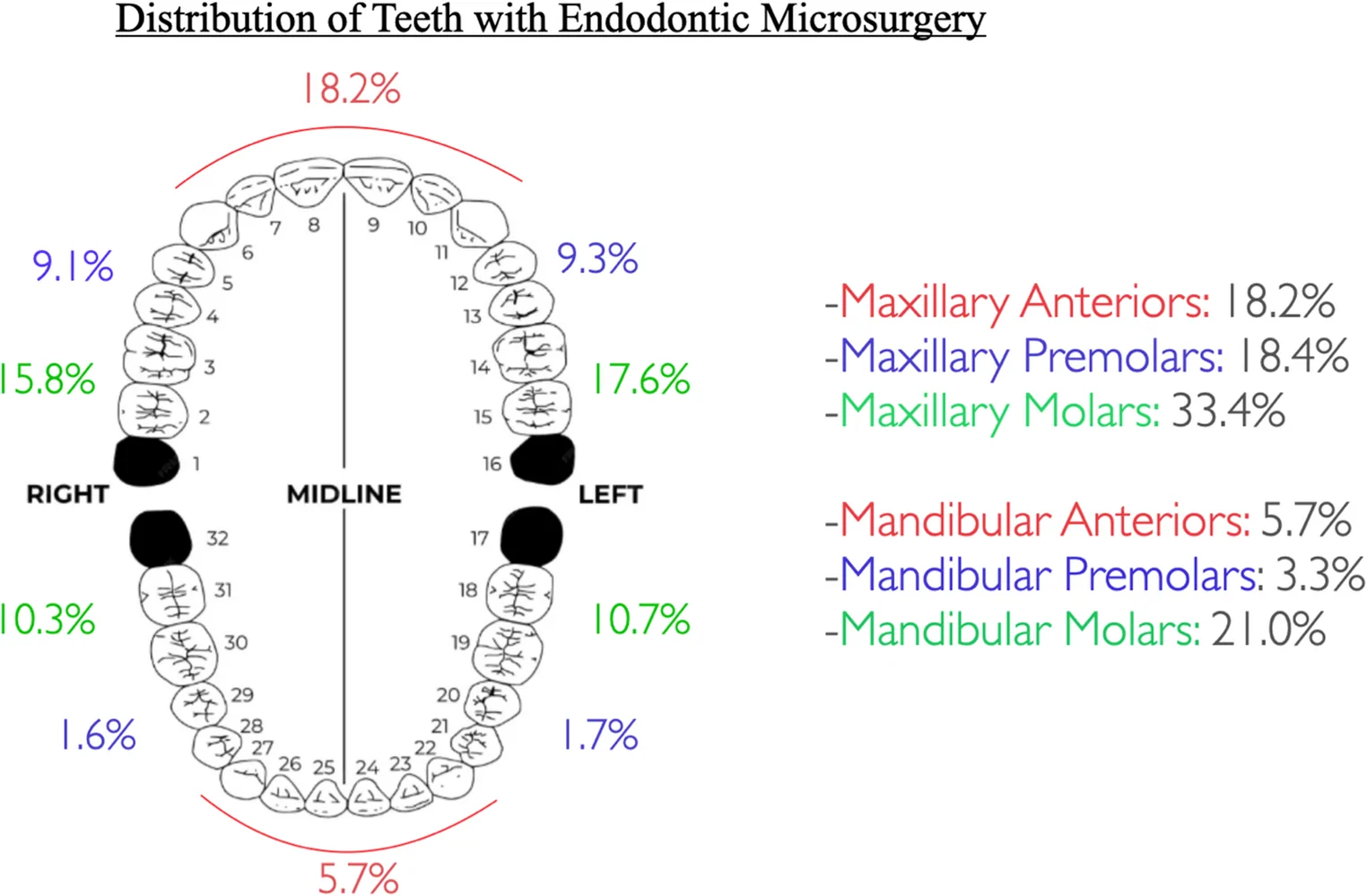
Distribution of EMS-treated teeth by arch and tooth class, with quadrant-level detail for premolars and molars. Values are counts; percentages (where shown) are of the total cohort (*n* = 1,672). Total (*n*) per tooth type include: Maxillary anteriors (306), maxillary premolars (305), maxillary molars (559), mandibular anteriors (97), Mandibular premolars (55), and mandibular molars (350); Teeth (*n*) per quadrant sections: Upper right premolars (151), upper right molars (265), upper left premolars (154), upper left molars (294), lower left premolars (28), lower left molars (180), lower right premolars (27) and lower right molars (170)

The top three indications within each tooth category are summarized in Table 4 (percentages calculated within category). Detailed values are not repeated here; briefly, large posts predominated in anterior and premolar teeth, whereas in molars uncleaned accessory canal and excessive calcification were most common.

The analysis for the BLT and ALT groups was conducted across all 15 reasons. For the purpose of the second study aim, the focus was on uncleaned accessory canal and excessive calcification, as these are the indications where the use of LAI during non-surgical treatment could potentially impact the outcome.

The number of occurrences for each reason for endodontic microsurgery (*n* = 1672), ranked from most to least frequent (Table 2), were as follows: large post (*n* = 372, 22.2%), uncleaned accessory canal (*n* = 364, 21.8%), excessive calcification (*n* = 240, 14.4%), apical cyst (*n* = 206, 12.3%), transportation/perforation (*n* = 143, 8.6%), unknown etiology (*n* = 125, 7.5%), external root resorption (*n* = 107, 6.4%), extrusion (*n* = 63, 3.8%), extraradicular pathosis/actinomycosis (*n* = 46, 2.8%), separated instrument (*n* = 43, 2.6%), root fracture (*n* = 37, 2.2%), failed apical surgery (*n* = 32, 1.9%), endodontic-periodontic infection (*n* = 5, 0.3%), apical cementicle (*n* = 4, 0.2%), and palatal gingival groove (*n* = 1, < 0.1%).

For the BLT group (*n* = 767), the number of occurrences per reason, ranked from most to least frequent, was as follows: uncleaned accessory canal (*n* = 204), large post (*n* = 170), apical cyst (*n* = 125), excessive calcification (*n* = 83), transportation/perforation (*n* = 55), external root resorption (*n* = 51), unknown etiology (*n* = 38), extrusion (*n* = 29), separated instrument (*n* = 28), extraradicular pathosis/actinomycosis (*n* = 23), root fracture (*n* = 22), failed apical surgery (*n* = 12), endodontic-periodontic infection (*n* = 4), apical cementicle (*n* = 1), and palatal gingival groove (*n* = 0).

For the ALT group (*n* = 905), the number of occurrences per reason, ranked from most to least frequent, was as follows: large post (*n* = 202), uncleaned accessory canal (*n* = 160), excessive calcification (*n* = 157), transportation/perforation (*n* = 88), unknown etiology (*n* = 87), apical cyst (*n* = 81), external root resorption (*n* = 56), extrusion (*n* = 34), extraradicular pathosis/actinomycosis (*n* = 23), failed apical surgery (*n* = 20), root fracture (*n* = 15), separated instrument (*n* = 15), apical cementicle (*n* = 3), endodontic-periodontic infection (*n* = 1), and palatal gingival groove (*n* = 1).

Compared with BLT, the ALT period showed a 8.9-percentage-point reduction in surgeries for uncleaned accessory canals (26.6% to 17.7%; 95% CI − 12.9 to − 4.9; *P* < .001) and a 6.5-percentage-point increase in surgeries for excessive calcification (10.8% to 17.3%; 95% CI + 3.2 to + 9.8; *P* < .001) (Fig. 4B).

**Fig. 4:**
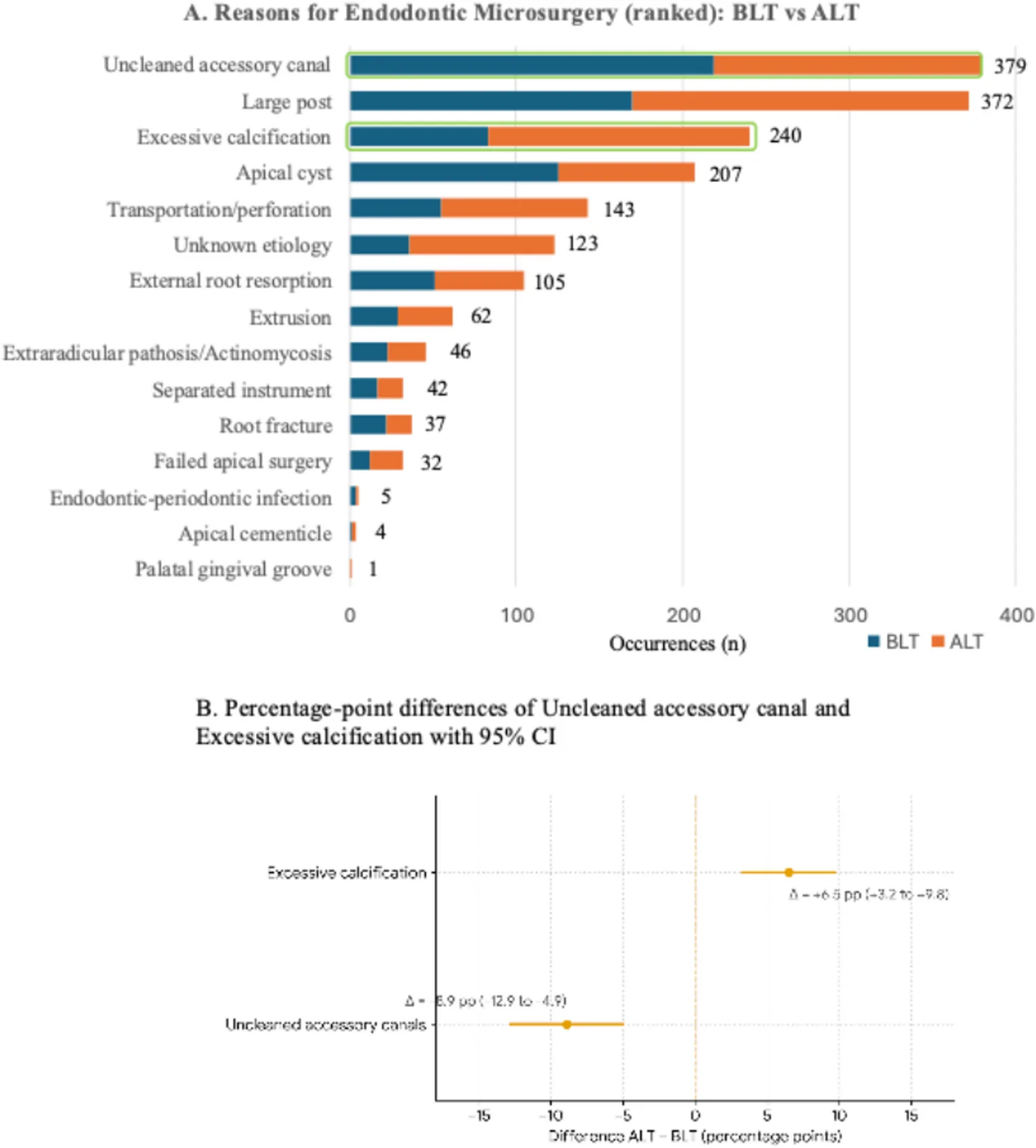
(**A**) Reasons for endodontic microsurgery (EMS) ranked by total occurrences (*n* = 1,672 teeth), with BLT and ALT distributions shown as stacked segments. Indications are ordered top-to-bottom by overall frequency. Highlighted in green are the two indications potentially influenced by laser-activated irrigation (LAI) during nonsurgical treatment: uncleaned accessory canal (*n* = 364) and excessive calcification (*n* = 240). Surgeries for uncleaned accessory canals decreased from 26.6% to 17.7%, whereas surgeries for excessive calcification increased from 10.8% to 17.3%. (**B**) Percentage-point differences (ALT − BLT) with 95% confidence intervals (95% CI): uncleaned accessory canals − 8.9 (− 12.9 to − 4.9); excessive calcification + 6.5 (+ 3.2 to + 9.8)

Negative-control indications showed no material period change, with Large posts remaining stable (22.2% [170/767] in BLT vs. 22.3% [202/905] in ALT; difference + 0.1% points; 95% CI − 3.9 to + 4.1) and Root fractures showing a negligible difference (2.9% [22/767] in BLT vs. 1.7% [15/905] in ALT; difference − 1.2% points; 95% CI − 2.7 to + 0.3), suggesting that not all indications fluctuated temporally during the study period. In the multivariable GEE models adjusting for patient demographics, tooth class, surgeon, and site, the ALT period remained significantly associated with lower odds of uncleaned accessory canal assignment (adjusted OR = 0.49; 95% CI 0.37 to 0.65; *P* < .001) and higher odds of excessive calcification assignment (adjusted OR = 1.65; 95% CI 1.21 to 2.25; *P* = .002) among EMS cases (Table 3). These adjusted findings indicate the temporal shifts were not explained by changes in patient clustering, surgeon, or site mix. To ensure these findings were not biased by procedure types unrelated to the intracanal irrigation protocol (e.g., resorption repairs or root amputations), a sensitivity analysis was performed restricting the GEE model exclusively to the clinically homogeneous subset of root-end apicoectomies (*n* = 1,573). Within this apicoectomy-only subset, the ALT period remained significantly associated with lower odds of uncleaned accessory canal assignment (adjusted OR = 0.46; 95% CI 0.35 to 0.62; *P* < .001) and higher odds of excessive calcification assignment (adjusted OR = 1.62; 95% CI 1.18 to 2.22; *P* = .003), confirming the stability of the primary analysis.

## Discussion

EMS is a reliable treatment option with favorable reported success and survival rates [1, 5]. Indications for EMS have been described in the literature, often focusing on isolated reasons for surgery. However, there is a notable lack of large-scale studies from private practice cohorts that comprehensively rank all indications. We did not enumerate or classify all non-surgical endodontic treatments (~ 22,000 cases) because doing so would not alter the within-EMS distributions or BLT–ALT contrasts; to mitigate secular-trend concerns, we included negative-control indications and surgeon/site-adjusted analyses, which yielded consistent results.

In this study of 1,672 teeth treated with EMS, the most common reasons were large posts (22.2%), uncleaned accessory canals (21.8%), and excessive calcification (14.4%). Large posts present a particular challenge because attempts at removal during retreatment may compromise root integrity and increase the risk of fracture, rendering the tooth non-restorable or prone to reinfection [16]. Nevertheless, the decision to forgo retreatment and proceed with EMS in these situations remains partly subjective, as the precise risk of fracture during post removal cannot be fully determined as there are other factors such as post size, length, and type (threaded, non-threaded, cast, flexi, para, fibre, etc.). Accessory canals—including delta canals, lateral canals, and isthmi—are another frequent indication, as they are often difficult to completely debride during non-surgical treatment [17, 18]. Similarly, excessive calcification, particularly pulp canal obliteration (PCO), poses a significant anatomic barrier that increases the risk of procedural errors such as perforation or instrument separation, making EMS the more predictable treatment option where patency cannot be achieved [19, 20]. Therefore, there are reasonable explanations why these three indications ranked highest in our cohort.

Less common indications included failed apical surgery (1.9%), endodontic-periodontic infections (0.3%), apical cementicles (0.2%), and palatal gingival groove (< 0.1%). Reported causes of failure after initial apical surgery include beveled root resections that expose infected dentinal tubules, leakage or dislodgement of retrofilling materials, and untreated isthmi or accessory canals not addressed during the first procedure. Modern microsurgery techniques and imaging have substantially improved outcomes in these resurgery scenarios, with success rates approaching those of primary microsurgery [21, 22]. In this cohort, failed apical surgery accounted for 1.9% of assigned indications. The initial surgeries for this population were largely performed during the era of modern endodontic microsurgery, a clinical paradigm broadly associated with high success rates in the literature [21–23]. However, because our study lacks a longitudinal denominator of all prior surgeries, we cannot estimate true surgical failure rates or definitively conclude that this low proportion reflects improved longitudinal outcomes.

Our study showed a high frequency of post-related indications in the maxillary arch, particularly maxillary anterior teeth (140/306, 45.8%) and maxillary premolars (120/305, 39.3%). This distribution is consistent with radiographic surveys reporting that posts are placed more often in the maxilla—especially in maxillary anterior and premolar teeth [24]. In addition, uncleaned accessory canals were common indications in both maxillary molars (150/559, 26.8%) and mandibular molars (152/350, 43.4%), likely reflecting the high prevalence of isthmus anatomy—between mesiobuccal-1 and mesiobuccal-2 canals in maxillary molars and between the mesiobuccal and mesiolingual canals in mandibular molars—which complicates complete debridement during nonsurgical treatment [17, 18].

To our knowledge, no prior clinical investigations have directly evaluated whether routine use of LAI influences the subsequent incidence of EMS. Existing research has focused primarily on antimicrobial efficacy, smear-layer and debris removal, or short-term clinical outcomes such as postoperative pain, rather than on long-term surgical incidence [11, 15]. The present study design represents a time-period comparison of surgical indications rather than a cause-and-effect analysis at the level of individual teeth. Moreover, separated instrument and root fracture—indications unlikely to be influenced by LAI—showed no period-related change, supporting the specificity of the uncleaned accessory canal association. The rise in calcification is addressed separately below through a biologic-lag interpretation. Together, these analyses strengthen the overall robustness of our findings. Nevertheless, the results remain associational and warrant confirmation in prospective, tooth-level studies. A primary limitation of this study is that LAI exposure was not measured at the individual-tooth level. Because BLT and ALT assignment relied strictly on the date of surgery, there is inherent temporal misclassification; teeth operated on during the ALT period may have received index treatments prior to LAI adoption or at external practices utilizing conventional irrigation. Consequently, this study serves as an ecological calendar-period comparison rather than a direct evaluation of LAI effectiveness. Furthermore, because exposure was assigned solely by calendar date, the exact rate of strict protocol adherence, individual irrigant volumes, and the number of teeth receiving early or selective LAI prior to the formal practice-wide adoption date cannot be explicitly quantified. Additionally, because the reviewing endodontists were not blinded to the calendar period during chart review, and because formal inter-rater reliability statistics were not calculated, there is a potential risk for differential classification of subjective intraoperative indications. Finally, due to the retrospective private-practice setting, the exact proportion of overall cases yielding successful biopsy specimens could not be explicitly quantified. Because diagnoses such as apical cyst and *Actinomycosis* were strictly dependent on definitive histopathologic confirmation, the reported frequencies of these specific indications may underestimate their true prevalence within the cohort.

The EdgePRO™ system is an Er, Cr: YSGG laser device (2780 nm) designed for intracanal disinfection during root canal treatment with LAI. Its mechanism of action involves rapid heating and vaporization of the irrigant, which generates expanding and collapsing vapor bubbles. The collapse of these bubbles produces acoustic and shock waves that propagate through the canal system, thereby enhancing the removal of microbes and debris from complex root canal anatomy. Among the 15 indications for EMS, the two most likely to be influenced by LAI are primarily, uncleaned accessory canal and secondarily, excessive calcification. The impact of LAI on cleaning accessory anatomy (lateral canals, isthmi, apical ramifications) is supported by multiple studies [11–14]. While LAI does not directly remove calcified dentin, the enhanced irrigant penetration and hydrodynamic agitation—together with improved smear-layer/debris removal in constricted canal portions—may secondarily assist canal negotiation in some cases by revealing micro-anatomic pathways that instruments can subsequently follow. In our study, the proportion of EMS cases performed for uncleaned accessory canals significantly decreased from 26.6% in the BLT group to 17.7% in the ALT group. This reduction indicates that untreated accessory anatomy constituted a significantly smaller proportion of the surgical caseload after LAI became routinely available in the practice.

In contrast, the proportion of EMS performed for excessive calcification increased from 10.8% in the BLT period to 17.3% in the ALT period. This pattern is biologically plausible as LAI does not mechanically remove calcified dentin and has limited direct effect on hard calcific obstructions. The increase, however, is noteworthy. Because pulp-canal calcifications are known to increase with patient age [25], we evaluated age distribution. While mean and median ages were descriptively similar between periods, aggregate metrics alone do not exclude age-related confounding. Therefore, patient age was adjusted for directly in our multivariable GEE models, which confirmed that the period-level shift in calcification was independent of age distribution. More plausible explanations for this shift include changes in external referral patterns, altered clinician thresholds for surgery versus nonsurgical retreatment, delayed presentation due to pandemic-related treatment backlogs, and improvements in diagnostic detection. Furthermore, because outcome categories in this study are compositional, a significant decline in the proportion of one major indication (such as uncleaned accessory canals) naturally increases the relative mathematical share of other indications. As a strictly hypothesis-generating possibility, this temporal increase could also partially reflect a lagged rise in pulp canal obliteration (PCO) associated with parafunctional loading during the COVID-19 pandemic. Chronic functional stress has been linked to pulpal calcification [26, 27], and pandemic-era reports document increased cracked teeth consistent with elevated bruxism and deferred care [28]. Notably, PCO is typically recognized ≥ 1 year after the inciting insult and can progress over multiple years [29, 30]. However, this causal pathway was not evaluated in our cohort and remains speculative.

In our study, apical cysts accounted for 12.3% (*n* = 206) of EMS cases. A landmark histopathologic study by Nair et al. examined 256 extracted teeth with persistent apical periodontitis and reported that 15% were periapical cysts, of which 9% were true cysts and 6% were pocket cysts [31]. True cysts are self-sustaining lesions that may persist despite adequate nonsurgical treatment, whereas pocket cysts are usually dependent on intraradicular infection and may resolve following conventional root canal therapy. A key limitation of surgical biopsy specimens is that histopathology cannot reliably distinguish between true and pocket cysts, because once the lesion is removed, its anatomic relationship to the root canal system is lost and the pathologist can only evaluate the cystic sac in isolation. Nair’s study overcame this limitation by examining extracted teeth with the periapical tissues intact, preserving the continuity between the root canal system and the lesion and allowing for more definitive classification. This diagnostic limitation highlights the complexity of assigning a single definitive etiology to apical cysts and underscores the need to interpret biopsy findings in the broader clinical and radiographic context. Accordingly, the 12.3% proportion of apical cysts in our cohort is likely more comparable to the overall 15% reported by Nair et al. than to the 9% proportion of true cysts alone. The proportion of true apical cysts within our cohort cannot be determined, as histopathologic analysis alone does not allow this distinction.

In this study, “unknown etiology” denotes cases in which recognized indications (e.g., extraradicular infection, cystic pathosis, fractures, procedural mishaps, or identifiable untreated anatomy) were not evident, yet symptoms and/or the periapical radiolucency persisted. The literature describes non-infectious mechanisms that can sustain periapical inflammation despite adequate orthograde treatment, notably cholesterol crystal aggregates that elicit a foreign-body giant-cell response and, more rarely, true foreign-body reactions to endogenous or exogenous material (e.g., sealer components). These processes are seldom distinguishable without surgical access and histopathologic examination, which explains why some clinically “unknown” cases proceed to EMS after conservative options are exhausted [7, 32, 33]. Finally, the findings of this study should be interpreted within the context of evolving clinical techniques, where modern irrigation and activation strategies increasingly aim to move beyond simple disinfection toward comprehensive dentin substrate conditioning [34]. Furthermore, as surgical techniques advance, the integration of regenerative biomaterials continues to expand the therapeutic limits and healing potential of endodontic surgery [35].

## Conclusion

In this large private practice cohort of 1,672 EMS cases, among 15 different possible indications, the most frequent were uncleaned accessory canal, post-related challenges, and excessive calcification. The proportion of uncleaned accessory canal-related surgeries significantly decreased in the period following the routine adoption of LAI. In contrast, the relative proportion of calcification-related surgeries was higher in the later period, the exact cause of which remains multifactorial and undetermined by this study design. Other uncommon indications, such as apical cementicles, palatal gingival groove, and endodontic-periodontic infections, remain infrequent and are unlikely to be influenced by advances in EMS. Overall, the distribution of recorded EMS indications differed significantly between the two calendar periods. However, causal explanations for these temporal shifts, including any potential adjunctive role of LAI, cannot be established by this study design. Determining whether LAI genuinely influences the downstream probability of requiring EMS requires a denominator-based longitudinal study with explicitly documented exposure.

## Data Availability

De-identified data is available from the corresponding author upon reasonable request.

## References

[1] Setzer FC (2022) Present status and future directions: surgical endodontics. Int Endod J 55:16–34 10.1111/iej.13783 35670053

[2] Setzer FC, Walter C, Sharma P, Petkovic-Curcin A, Kim S (2019) Outcome of crown and root resection: a systematic review. J Endod 45:13–19 10.1016/j.joen.2018.10.003 30527594

[3] Wu SY, Cheng HL, Chen YH, Hsieh YD (2021) A long-term treatment outcome of intentional replantation: modern technique, viability and survival. J Endod 47:1234–1242

[4] Saito T, Ogiso B, Sato T, Takahashi S (2020) Treatment of inflammatory external root resorption with endodontic microsurgery, apicoectomy, and mineral trioxide aggregate restoration: a case report. Jpn Endod Assoc J 41:193–197

[5] Setzer FC, Shah SB, Kohli MR, Karabucak B, Kim S (2010) Outcome of endodontic surgery: a meta-analysis—Part 1: comparison of traditional root-end surgery and endodontic microsurgery. J Endod 36:1757–1765 10.1016/j.joen.2010.08.007 20951283

[6] Tortorici S, Difalco P, Caradonna L, Tetè S (2014) Traditional endodontic surgery versus modern technique: a 5-year controlled clinical trial. J Craniofac Surg 25:804–807 10.1097/SCS.0000000000000398 24469371

[7] Ricucci D, Siqueira JF Jr, Rôças IN (2015) Extraradicular infection as the cause of persistent symptoms: a case series. J Endod 41:265–273 10.1016/j.joen.2014.08.020 25282379

[8] von Arx T, Hänni S (2015) Clinical and radiographic assessment of predictors for healing one year after periapical surgery. J Endod 41:589–598 10.1016/j.joen.2006.10.001 17258628

[9] Tawil PZ, Trope M (2020) The endodontic–restorative connection: a 15-year systematic review of longitudinal clinical studies. J Endod 46(5S):S2–S13

[10] Venskutonis T, Plotino G, Tocci L, Gambarini G (2014) Periapical and endodontic status scale using cone-beam computed tomography. J Endod 40:652–658 10.1016/j.joen.2014.10.017 25498834

[11] DiVito E, Peters OA, Peters CI (2014) Effect of laser-activated irrigation on smear layer and debris removal: a scanning electron microscope study. J Endod 40:151–154

[12] Fahim SZ, Ghali RM, Hashem AA, Farid MM (2024) The efficacy of 2780 nm Er,Cr;YSGG and 940 nm Diode Laser in root canal disinfection: A randomized clinical trial. Clin Oral Investig 28(3):175 10.1007/s00784-024-05563-z PMC10894764 38403667

[13] Suter B, Suter V, Lussi A (2019) The effectiveness of laser-activated irrigation compared with conventional irrigation in root canal disinfection: a systematic review. Int Endod J 52:1663–1675

[14] Meire MA, De Moor RJG (2024) Principle and antimicrobial efficacy of laser-activated irrigation: A narrative review. Int Endod J 57(7):841–860 10.1111/iej.14042 38340037

[15] Sabeti M, Harouni A, Gabbay J (2026) Comparing Ultrasonically Activated Irrigation and Laser-Activated Irrigation for Postoperative Pain Reduction in Endodontics: A Systematic Review and Meta-Analysis of Randomized Controlled. Trials. J Endod 52:37–46 10.1016/j.joen.2025.08.002 40818665

[16] Abramovitz I, Better H, Shacham A, Shlomi B, Metzger Z (2002) Case selection for apical surgery: a retrospective evaluation of associated factors and rationale. J Endod 28:527–530 10.1097/00004770-200207000-00010 12126382

[17] Ricucci D, Siqueira JF Jr (2010) Fate of the tissue in lateral canals and apical ramifications in response to pathologic conditions and treatment procedures. J Endod 36:1–15 10.1016/j.joen.2009.09.038 20003929

[18] Vertucci FJ (2005) Root canal morphology and its relationship to endodontic procedures. Endod Top 10:3–29

[19] Zuolo ML, Ferreira MO, Gutmann JL (2005) Prognosis in periradicular surgery: a meta-analysis. Int Endod J 38:521–526 10.1046/j.1365-2591.2000.00263.x 11307456

[20] Tavares PM, Cândido MS, Souza E et al (2020) Surgical management of endodontic failures due to calcifications and technical difficulties. Dent Press Endod 10:52–61

[21] Kim D, Ku H, Nam T, Shin SJ, Kim E (2018) Outcome of endodontic micro-resurgery: a retrospective study with propensity-matched comparison to primary microsurgery. J Endod 44:183–189 10.1016/j.joen.2018.07.024 30243664

[22] Taschieri S, Del Fabbro M, Testori T, Francetti L, Weinstein RL (2007) Endodontic reoperation using an endoscope and microsurgical instruments: a prospective clinical study. Int Endod J 40:186–193

[23] Kim S, Kratchman S (2006) Modern endodontic surgery concepts and practice: a review. J Endod 32:601–623 10.1016/j.joen.2005.12.010 16793466

[24] Almaghrabi J, Alesawi A, Attar E, Alshali S (2022) Radiographic analysis of posts performed by undergraduate dental students: a cross-sectional study. Clin Cosmet Investig Dent 14:37–43 10.2147/CCIDE.S337012 PMC8799894 35115842

[25] Kiefner P, Connert T (2017) Treatment of calcified root canals in older people. Gerodontology 34:285–292 10.1111/ger.12238 27296318

[26] Malhotra N, Mala K (2013) Calcific metamorphosis: literature review and clinical strategies. Dent Update 40:48–60 10.12968/denu.2013.40.1.48 23505858

[27] Farias Z, Sousa J, Faria C, Vieira J, Sobral A, Silveira M (2023) Pulpal calcifications in orthodontically moved teeth: a scoping review. J Clin Exp Dent 15:e773–e780 10.4317/jced.60777 PMC10550074 37799748

[28] Nosrat A, Yu P, Verma P, Dianat O, Wu D, Fouad AF (2022) Was the COVID-19 pandemic associated with an increased rate of cracked teeth? J Endod 48:1241–1247 10.1016/j.joen.2022.07.002 PMC9273286 35835260

[29] McCabe PS, Dummer PMH (2012) Pulp canal obliteration: an endodontic diagnosis and treatment challenge. Int Endod J 45:177–197 10.1111/j.1365-2591.2011.01963.x 21999441

[30] Andreasen FM, Zhijie Y, Thomsen BL, Andersen PK (1987) Occurrence of pulp canal obliteration after luxation injuries in the permanent dentition. Endod Dent Traumatol 3:103–115 10.1111/j.1600-9657.1987.tb00611.x 3476298

[31] Nair PN, Pajarola G, Luder HU (1996) Types and incidence of human periapical lesions obtained with extracted teeth. Oral Surg Oral Med Oral Pathol Oral Radiol Endod 81:93–102 10.1016/s1079-2104(96)80156-9 8850492

[32] Duncan HF et al (2023) Treatment of pulpal and apical disease: The European Society of Endodontology (ESE) S3-level clinical practice guideline. Int Endod J 56:239–314 10.1111/iej.13974 37772327

[33] Torabinejad M, Corr R, Handysides R, Shabahang S (2009) Outcomes of nonsurgical retreatment and endodontic surgery: a systematic review. J Endod 35:930–937 10.1016/j.joen.2009.04.023 19567310

[34] Riccitiello F et al (2026) Modern endodontic irrigation and activation: From disinfection to dentin substrate conditioning—A narrative review. Appl Sci 16:4593

[35] di Lauro AE et al (2023) The effectiveness of autologous platelet concentrates in the clinical and radiographic healing after endodontic surgery: A systematic review. Materials 16(22):7187 10.3390/ma16227187 PMC10672445 38005117

